# Genetic Recombination between Human and Animal Parasites Creates Novel Strains of Human Pathogen

**DOI:** 10.1371/journal.pntd.0003665

**Published:** 2015-03-27

**Authors:** Wendy Gibson, Lori Peacock, Vanessa Ferris, Katrin Fischer, Jennifer Livingstone, James Thomas, Mick Bailey

**Affiliations:** 1 School of Biological Sciences University of Bristol, Bristol, United Kingdom; 2 Department of Clinical Veterinary Science, University of Bristol, Langford, Bristol, United Kingdom; Lancaster University, UNITED KINGDOM

## Abstract

Genetic recombination between pathogens derived from humans and livestock has the potential to create novel pathogen strains, highlighted by the influenza pandemic H1N1/09, which was derived from a re-assortment of swine, avian and human influenza A viruses. Here we investigated whether genetic recombination between subspecies of the protozoan parasite, *Trypanosoma brucei*, from humans and animals can generate new strains of human pathogen, *T*. *b*. *rhodesiense* (*Tbr*) responsible for sleeping sickness (Human African Trypanosomiasis, HAT) in East Africa. The trait of human infectivity in *Tbr* is conferred by a single gene, *SRA*, which is potentially transferable to the animal pathogen *Tbb* by sexual reproduction. We tracked the inheritance of *SRA* in crosses of *Tbr* and *Tbb* set up by co-transmitting genetically-engineered fluorescent parental trypanosome lines through tsetse flies. *SRA* was readily transferred into new genetic backgrounds by sexual reproduction between *Tbr* and *Tbb*, thus creating new strains of the human pathogen, *Tbr*. There was no evidence of diminished growth or transmissibility of hybrid trypanosomes carrying *SRA*. Although expression of *SRA* is critical to survival of *Tbr* in the human host, we show that the gene exists as a single copy in a representative collection of *Tbr* strains. *SRA* was found on one homologue of chromosome IV in the majority of *Tbr* isolates examined, but some Ugandan *Tbr* had *SRA* on both homologues. The mobility of *SRA* by genetic recombination readily explains the observed genetic variability of *Tbr* in East Africa. We conclude that new strains of the human pathogen *Tbr* are being generated continuously by recombination with the much larger pool of animal-infective trypanosomes. Such novel recombinants present a risk for future outbreaks of HAT.

## Author Summary

Genetic recombination allows transfer of harmful traits between different strains of the same pathogen and enables the emergence of genetically novel pathogen strains that the host population has not previously encountered. This can be particularly important when a pathogen acquires a virulence trait that allows it to spread beyond its normal host population. Here we show that this happens among the single-celled parasites—trypanosomes—that cause human African trypanosomiasis (HAT) or sleeping sickness carried by the tsetse fly. Genetic recombination readily occurs between the human and animal parasites when they are co-transmitted by the tsetse fly, creating new pathogen genotypes or strains. There is a single gene that confers human infectivity and each of the genotypes that inherits this gene is potentially capable of infecting humans. In this way new strains of the human pathogen can be generated by recombination between the human-infective and animal-infective trypanosomes. Such novel recombinants present a risk for future outbreaks of HAT.

## Introduction

Genetic recombination can generate new pathogen strains to which host populations have no prior immunity. This can have disastrous consequences; for example, the human population is at risk of an influenza pandemic caused by recombination between viruses derived from humans and domestic livestock. Microbial genetic recombination facilitates the transfer of genes for virulence and drug resistance into new genetic backgrounds, potentially creating pathogen strains with novel phenotypes as well as accelerating the spread of drug resistance. Among eukaryote pathogens, the impact of sexual reproduction is hard to predict, because of the wholesale mixing of genes from different strains.

*Trypanosoma brucei* is the protist parasite responsible for the vector-borne disease human African trypanosomiasis (HAT) or sleeping sickness. In East Africa the disease is a zoonosis caused by *T*. *b*. *rhodesiense* (*Tbr*) which is morphologically indistinguishable from the non-human infective subspecies, *T*. *b*. *brucei* (*Tbb*). Both subspecies may occur in the same range of wild or domestic mammalian hosts and there has been a long-standing controversy about their identification [1]. This was resolved by the discovery that human infectivity in *Tbr* was governed by expression of a single gene (Serum Resistance Associated, *SRA*) [2] and the presence of the *SRA* gene now serves as a convenient marker for *Tbr* [3–5].

Clearly, transfer of this single gene could potentially generate new strains of human-infective trypanosomes, and this has been demonstrated experimentally by transfection of the *SRA* gene into *Tbb*, resulting in a trypanosome with a human-infective phenotype [2]. Population genetics analyses have failed to find consistent genotypic differences between *Tbr* and *Tbb*, other than presence/absence of *SRA*, and the idea that *Tbb* and *Tbr* are freely interchangeable by transfer of *SRA* has become central to the interpretation of population genetics data for *Tbr* and *Tbb* [6]; evidence of genetic admixture between *Tbr* and *Tbb* from recent genome comparisons of the two subspecies also supports this interpretation [7,8]. Genetic exchange in *T*. *brucei* occurs in the insect vector, the tsetse fly (genus *Glossina*) [9] and recent results show that it has the hallmarks of conventional eukaryote sexual reproduction: meiosis and production of haploid gametes [10,11]. All subspecies of *T*. *brucei*, including *Tbr*, have been shown to express meiosis-specific genes [11]. Genetic crosses between *Tbr* and *Tbb* have been carried out in the laboratory [12–14], but analysis of the progeny was carried out before the significance of *SRA* was recognised and presence/absence of the gene was not determined. Potential human infectivity of hybrid progeny was tested by analysing resistance to lysis by human serum [15]; however, this is not such a reliable test for human infectivity as presence of the *SRA* gene.

*SRA* appears to be a single copy gene that resides in one of the telomeric expression sites (ES) for variant surface glycoprotein (VSG) genes, such that, when this ES is transcribed, *SRA* is also expressed [2]. The ES containing *SRA* is unusually short in that it contains only three ES-associated genes (*ESAGs* 5, 6 and 7), with *SRA* located between *ESAG 5* and the telomeric *VSG* gene [2]. Both the *SRA* gene and its immediate genomic environment are conserved in different *Tbr* strains [16]. The chromosome carrying *SRA* has not been identified, though from its size (1.6 Mb [2]), it appears to be one of the smaller diploid chromosomes described in *T*. *brucei* [17]. It is also uncertain whether all *Tbr* strains carry only a single *SRA* allele or have multiple ES with *SRA*. It is technically difficult to sequence *T*. *brucei* ES because of their telomeric location [18], and the few studies to date show within-strain similarity of ES in structure and gene content [19–21], making it difficult to distinguish between different ES in the same trypanosome strain.

From an evolutionary perspective, it seems unlikely that *Tbr* would have only a single *SRA* gene, as that would make it dependent on only a single ES for infection in the human host; antigenic variation would be restricted to replacement of the *VSG* in this ES, and switching to expression of another ES, which lacked *SRA*, would be lethal for the parasite. Dependence on this one ES in the human host would lock the trypanosome into expression of the single transferrin receptor encoded by the *ESAG 6* and *ESAG 7*genes co-transcribed with *SRA* [22]. Moreover, according to the hypothesis that allelic variation in *ESAG 6* and *ESAG 7*is adaptive for uptake of different mammalian transferrins [23,24], the receptor encoded by alleles in the *SRA* ES should be specific for human transferrin. A further problem confronts the trypanosome on transmission from tsetse to human, because metacyclics, the infective forms inoculated with the fly’s saliva, express a restricted set of *VSG*s residing in specialized ES lacking *ESAG*s [25] and presumably also *SRA*. Without protection of the SRA protein to inactivate the trypanolytic effect of human serum, how is it possible for *Tbr* metacyclics to survive the transition from fly to human?

Here we provide the definitive experimental proof that *SRA* is readily transferred between *Tbr* and *Tbb* during sexual reproduction, creating new genotypes of the human pathogen *Tbr*, because the *SRA* gene is now in a new genetic background consisting of an equal mixture of the parental *Tbr* and *Tbb* genomes. We show that *SRA* is present as a single copy on one homologue of chromosome IV in the majority of *Tbr* strains analysed and explore the implications for the epidemiology of HAT in East Africa.

## Materials and Methods

### Ethics statement

Animal experiments were approved by the University of Bristol Ethical Review Group (Home Office licence PIL 30/1248) and carried out under the UK government Animals (Scientific Procedures) Act 1986.

### Trypanosomes and cell culture

The following tsetse-transmissible strains of *Trypanosoma brucei rhodesiense* (*Tbr*) and *T*. *b*. *brucei* (*Tbb*) were used: *Tbr* 058 (MHOM/ZM/74/58 [26,27]); *Tbr* LUMP 1198 (MHOM/UG/76/LUMP 1198 [26,27]); *Tbr* TOR11(MHOM/UG/88/TOR11 [28]); *Tbb* J10 (MCRO/ZM/73/J10 CLONE 1 [26,27]); *Tbb* 1738 (MOVS/KE/70/EATRO 1738 [27,29]); *Tbb* 427 (MOVS/UG/60/427 VAR3 [30]). These strains represent a range of *Tbr* and *Tbb* genotypes from East Africa; isolate details are in S1 Table. *Tbr* 058 and all three *Tbb* strains have proved mating-competent in previous crosses. The *Tbr* and *Tbb* clones carried cytoplasmically-expressed genes for enhanced green fluorescent protein (GFP) [31] or monomeric red fluorescent protein (RFP) [32,33], respectively.

Procyclic form (PF) trypanosomes were grown in Cunningham’s medium (CM) [34] supplemented with 10% v/v heat-inactivated foetal calf serum, 5 μg/ml hemin and 10 μg/ml gentamycin at 27°C. PF were transfected by electroporation as previously described [30] and clones were obtained by limiting dilution of PF in CM in 96 well plates incubated at 27°C in 5% CO_2_.

### Experimental crosses

**Fig 1:**
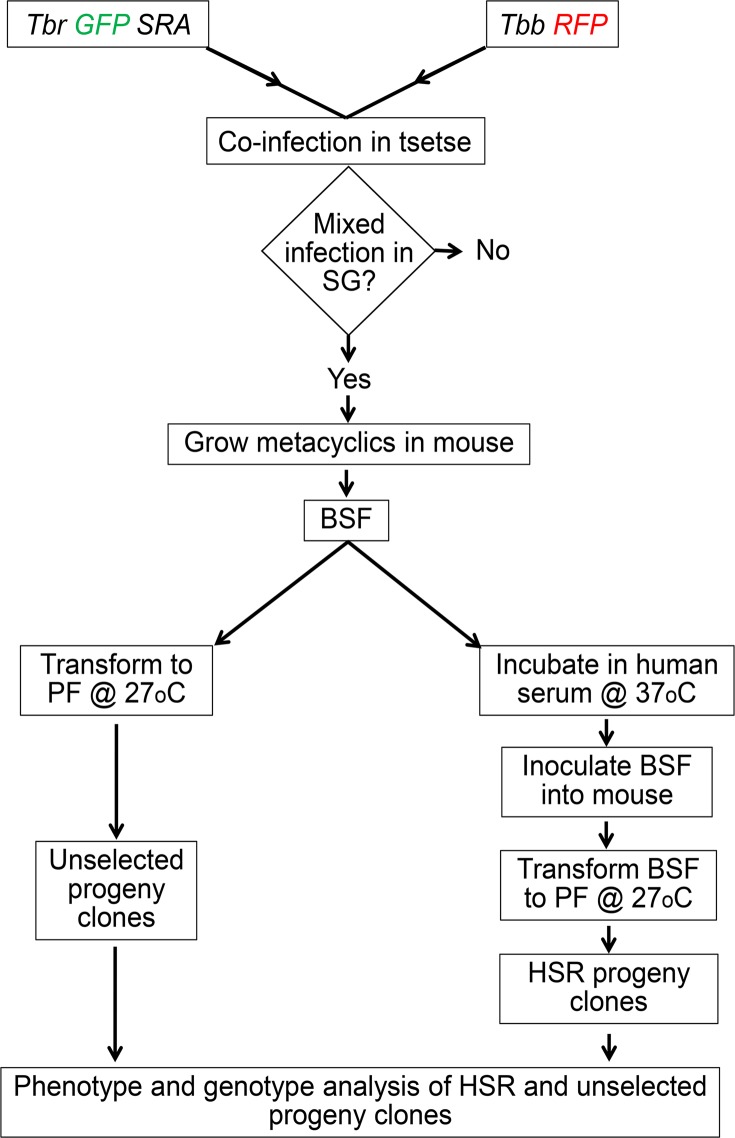
Flow chart of experimental design. Experimental crosses were carried out by co-transmitting green fluorescent *Tbr* and red fluorescent *Tbb* through tsetse. Salivary glands with a mixed infection of red and green fluorescent trypanosomes were taken forward for analysis. BSF, bloodstream forms; PF, procyclic forms; HSR, human serum resistant.

Nine pairwise crosses were carried out, each involving one *Tbr* GFP clone and one *Tbb* RFP clone (crosses 1–9, Table 1), such that hybrids carrying both fluorescent markers appear yellow [33]. Groups of 15–25 tsetse flies (*Glossina morsitans morsitans* or *G*. *pallidipes*) were infected on their first feed essentially as described previously [35,36]. The infective bloodmeal consisted of approximately 8 x 10^6^ bloodstream form (BSF) trypanosomes ml^-1^ in sterile horse blood (TCS Biosciences, UK), or approximately 10^7^ PF trypanosomes ml^-1^ of washed horse red blood cells resuspended in Hank’s Balanced Salt Solution, supplemented with 10mM L-glutathione [37]. Infected flies were maintained on sterile horse blood until dissection approximately 5 weeks following the infective feed. Salivary glands (SG) were dissected in a drop of phosphate buffered saline and examined for the presence of fluorescent trypanosomes using a DMRB microscope (Leica) equipped with a Retiga Exi camera (QImaging) and Volocity software (PerkinElmer). SG containing an approximately equal mixture of trypanosome clones as judged by fluorescence were taken forward for isolation of hybrids (Fig. 1).

**Table 1:** Genetic crosses of *T*. *b*. *rhodesiense* (*Tbr*) and *T*. *b*. *brucei* (*Tbb*).

Cross	*Tbr*	*Tbb*	No. of mixed SG analysed	No. of clones analysed^a^	No. of clones with SRA^a^	No. of hybrid genotypes^b^	No. of hybrid genotypes with SRA (%)
1	058 GFP	1738 RFP	6	67 (a. 47; b. 20)	59/67 (88%) a. 39/47 (83%) b. 20/20 (100%)	14	12 (86%)
2	058 GFP	427 var 3 RFP	4	39 (a. 33; b. 6)	35/39 (90%) a. 29/33 (88%) b. 6/6 (100%)	4	4 (100%)
3	058 GFP	J10 RFP	3	41 (a. 14; b. 27)	35/41 (85%) a. 12/14 (86%) b. 23/27 (85%)	7	7 (100%)
4	TOR11 GFP	1738 RFP	4	37 (a. 25; b. 12)	28/37 (76%) a. 16/25 (64%) b. 12/12 (100%)	7	7 (100%)
5	TOR11 GFP	427 var 3 RFP	3	21 (a. 12; b. 9)	13/21 (62%) a. 9/12 (75%) b. 4/9 (44%)	0	0
6	TOR11 GFP	J10 RFP	4	31 (a. 17; b. 14)	26/31 (84%) a. 14/17 (82%) b. 12/14 (86%)	10	10 (100%)
7	LUMP 1198 GFP	1738 RFP	2	18 (a. 11; b. 7)	11/18 (61%) a. 4/11 (36%) b. 7/7 (100%)	10	6 (60%)
8	LUMP 1198 GFP	427 var 3 RFP	3	23 (a. 12; b. 11)	22/23 (96%) a. 11/12 (92%) b. 11/11 (100%)	0	0
9	LUMP 1198 GFP	J10 RFP	4	28 (a. 13; b. 15)	23/28 (82%) a. 11/13 (85%) b. 12/15 (80%)	0	0
10^c^	058	TSW 196	2	14	Not selected	6	2 (33%)
11^c^	058H	KP2N	5	38	Not selected	13	11 (85%)

^a^ Population a. unselected; population b. selected with human serum. Not selected: populations not selected with human serum before cloning.

^b^ As the same hybrid genotype was sometimes recovered from both selected and unselected populations, populations a and b are not distinguished in this column.

^c^ Cross 10 described by [12]; cross 11 described by [56].

### Isolation and analysis of progeny clones

Metacyclics from infected SG were inoculated into mice (SCID or immunosuppressed MF1) and infected blood was harvested from the first peak of parasitaemia. Aliquots of approximately 10^7^ BSF cells in whole blood were (a) transformed directly to PF by incubation in CM at 27°C, or (b) incubated in HMI-9 medium [38] with heat-inactivated human serum (WG serum donor) for 24 hours at 37°C to select human serum resistant (HSR) parasites, followed by inoculation into a mouse (SCID) and subsequent transformation of BSF from the first peak of parasitaemia into PF as in (a) above (Fig. 1). Clones were obtained from populations (a) unselected and (b) HSR by limiting dilution as above (Table 1), and grown in CM for purification of DNA using a spin column DNA purification kit. Microsatellite analysis was performed as described [33,36] for between four and six loci per clone, depending on the allelic differences between the parental clones used for the cross[39][39]. The presence of the *SRA* gene was detected by PCR using primers SRA E (5’-TACTGTTGTTGTACCGCCGC) and SRA J (5’-GTACCTTGGCGCGCTCGCGCTG) followed by gel electrophoresis [27].

Samples for pulsed field gel (PFG) electrophoresis were prepared by lysing and deproteinising trypanosomes *in situ* in agarose blocks [40]. PFG electrophoresis, blotting and hybridization were carried out essentially as described [33] using PCR-amplified DNA fragments as specific probes for genes encoding SRA and DNA topoisomerase (TOPO; chromosome IV).

Kinetoplast DNA maxicircle type was determined for selected clones as previously described [33].

### Chromosomal location of *SRA*

Two approaches were used to identify the chromosomal location of *SRA*: (a) Quantitative PCR (qPCR) of *SRA* and chromosome-specific genes for chromosomes I-V (S2 Table). DNA was extracted from individual chromosome bands of *Tbr* 058 and LUMP 1198 after PFG chromosomal separation; gel bands were cut out and purified using GeneJet Gel Extraction Kit (Fermentas) according to manufacturer’s instructions for large chromosomes (>10kb DNA). All qPCRs were executed with 300nM primer concentrations (S2 Table) using a SYBR Green/ROX qPCR Master Mix (Fermentas) according to manufacturer’s instructions with 5 ng of template DNA per reaction; melting curve analysis was carried out to verify amplification of a single PCR product. Resulting data were analysed using MX Pro software (Agilent Technologies). (b) Sequential hybridisation of PFG blots with probes for various genes [β-tubulin (TUB), chromosome I; trypanothione synthetase (TS), chromosome II; paraflagellar rod protein (PFR1), chromosome III; DNA topoisomerase (TOPO), chromosome IV; lysosomal membrane protein (P67), chromosome V] was used to establish co-localisation with *SRA*. PFG samples were prepared from various *Tbr* isolates (S1 Table) and analysed as described above.

### Copy number of *SRA*

*SRA* copy number relative to the housekeeping gene encoding triose phosphate isomerase (TIM) was determined in a range of *Tbr* samples (S1 Table); *TIM* is present in two copies on homologous chromosomes [41]. QPCR was used to analyse the copy number of both genes and deduce the ratio of *SRA* to *TIM*, using SYBR-Green for detection and quantification of amplified DNA. QPCR conditions for amplification were optimized using a ten-fold dilution series of a plasmid construct containing one copy of each gene; after optimization, the nucleotide primers (S1 Fig) were used at 300nM *SRA* and 500nM *TIM* final concentration. All qPCR reactions were performed in triplicate and a positive control (with reference DNA) and a negative control (without DNA) were included in each set of reactions; qPCR reactions were run using a SYBR Green/ROX qPCR Master Mix (Fermentas) according to manufacturer’s instructions with 5 ng of template DNA per reaction; melting curve analysis was carried out to verify amplification of a single PCR product. Resulting data were analysed using MX Pro software (Agilent Technologies).

## Results

### Inheritance of human infectivity

**Fig 2:**
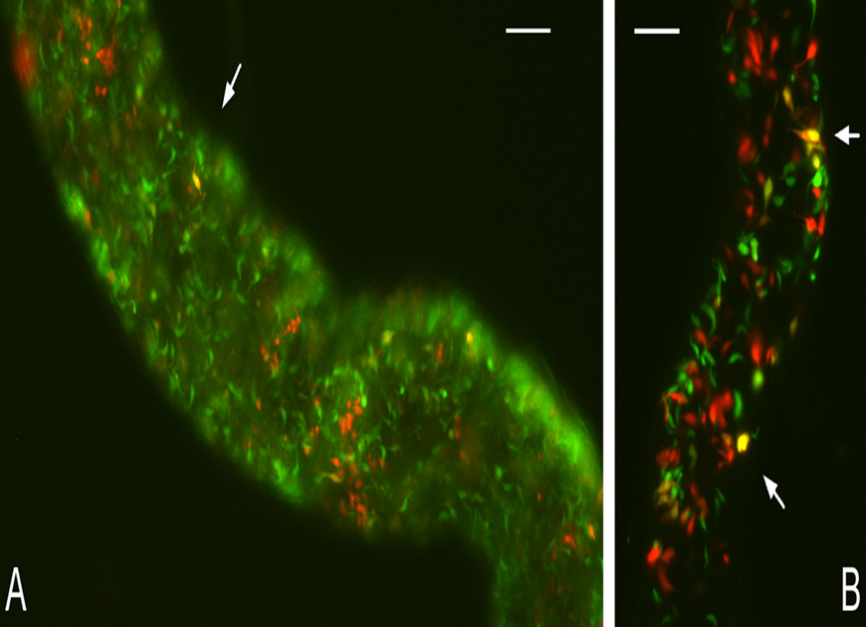
Yellow hybrids of *T*. *b*. *rhodesiense* and *T*. *b*. *brucei* in tsetse salivary glands. A. Part of salivary gland containing *T*. *b*. *rhodesiense* 058 GFP and *T*. *b*. *brucei* 1738 RFP. Arrows: yellow fluorescent trypanosomes. Scale bar 100 μm. B. Part of salivary gland containing *T*. *b*. *rhodesiense* LUMP 1198 GFP and *T*. *b*. *brucei* J10 RFP; despite the presence of yellow fluorescent trypanosomes (arrows), no hybrid trypanosomes were recovered from this cross. Scale bar 50 μm.

We set out to test whether genetic recombination between *Tbr* and *Tbb* enabled transfer of *SRA* into new genetic backgrounds and created potentially human infective hybrid genotypes. To detect hybrids we carried out pairwise crosses of three green fluorescent clones of *Tbr* with three red fluorescent strains of *Tbb* (crosses 1–9, Table 1), such that hybrids would appear yellow (Fig. 2) [33]. Each of the three *Tbr* strains successfully mated with at least one of the *Tbb* strains, as judged by the production of hybrid clones; no hybrid progeny were recovered from crosses 5, 8 and 9 (Table 1). Clones were isolated either before (population a, unselected) or after incubation with human serum (population b, selected) (Fig. 1). The majority of clones (252 of 305, 83%) had the *SRA* gene whether derived from the selected or unselected populations (Table 1), demonstrating that *SRA*+ trypanosomes were not outcompeted by *SRA*- trypanosomes during development in the fly or growth as BSF in the mouse. A few *SRA*- clones survived incubation with human serum (12 of 119, 10%), but the majority of human serum resistant clones had *SRA* (107 of 119, 90%). Each clone was genotyped by microsatellite and molecular karyotype analysis, and also, where informative, kinetoplast maxicircle DNA type. Some genotypes were represented by more than one clone and found in both the human serum selected and unselected populations. Of the hybrid genotypes recovered, over half carried the *SRA* gene (Table 1), confirming that this gene can be transferred into different genetic backgrounds by sexual reproduction. We also confirmed presence of the *SRA* gene in hybrid clones from two previous crosses of *Tbr* 058 (crosses 10 and 11, Table 1).

### Chromosomal location of *SRA*

**Fig 3:**
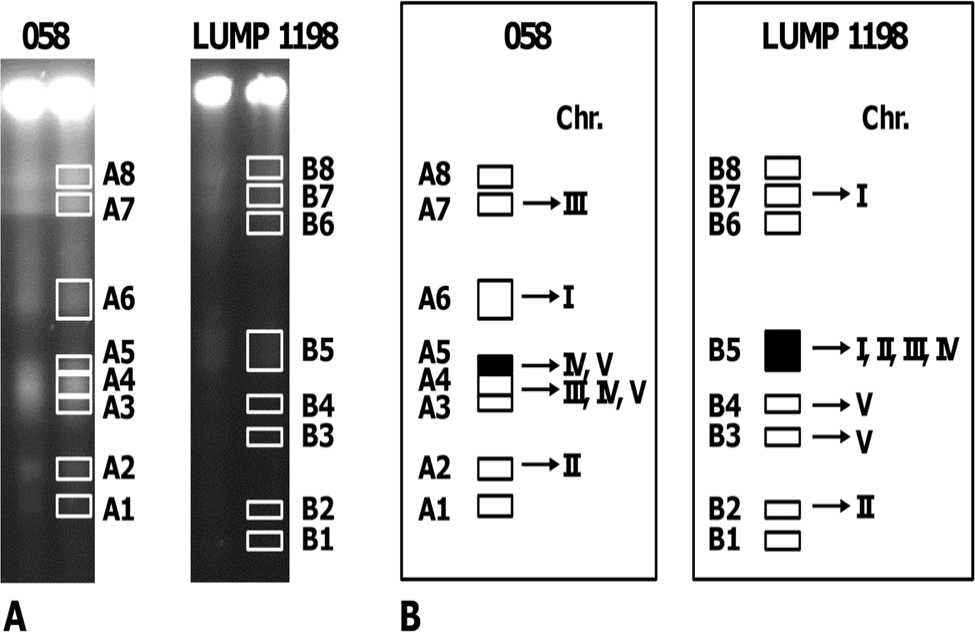
Chromosome location of *SRA* in *T*. *b*. *rhodesiense*. A. Chromosomal bands of *T*. *b*. *rhodesiense* 058 and LUMP 1198 separated by PFG; boxes outline the bands cut out and purified. B. Graphical summary of quantitative PCR results for individual chromosomal bands; the filled black boxes indicate the chromosome band with *SRA*. C_t_ values are given in Tables S3 and S4.

In previous analysis of another *Tbr* strain, ETat 1, there appeared to be only one copy of the *SRA* gene, residing in an unusual truncated *VSG* expression site (ES) that contained only three ES associated genes (ESAGs) [2]. In other *Tbr* isolates the local genomic environment of *SRA* was conserved [16], but there could be more than one copy of this ES and hence more than one copy of *SRA*. To investigate the chromosomal location of *SRA*, we purified DNA from individual chromosomal bands of *Tbr* 058 and LUMP 1198 and tested for the presence of various chromosome-specific genes (S2 Table) by qPCR. The C_t_ values for each gene tested are shown in Tables S3 and S4 and the results are shown graphically in Fig. 3. The lowest C_t_ value for *Tbr* 058 was for chromosomal band A5 corresponding to chromosomes IV and V, while that for *Tbr* LUMP 1198 was for chromosomal band B5 corresponding to chromosomes I–IV (Tables S3, S4 and Fig. 3). The combined results are consistent with the localisation of *SRA* to chromosome IV.

**Fig 4:**
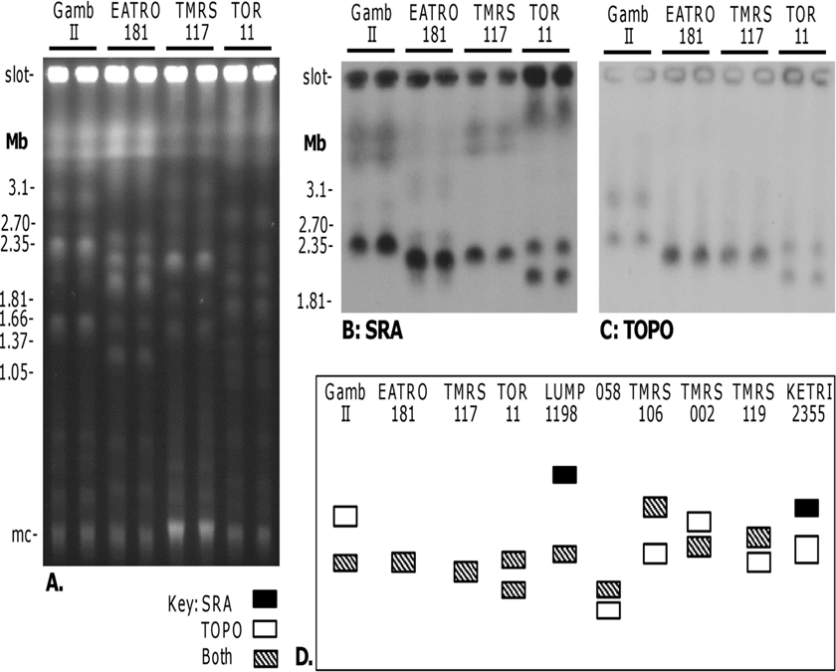
Co-localisation of *SRA* and *TOPO* genes. A. Ethidium bromide stained gel comparing the molecular karyotypes of clones from four strains of *T*. *b*. *rhodesiense* (*Tbr*). Size marker: chromosomal DNA from *Hansenula wingei*; mc = minichromosomes of 50–100 kb in size. B, C. Autoradiographs of blots of this PFG gel following hybridization with the probes indicated. Blots were washed to 0.1 × SSC at 65°C. D. Diagram of *SRA* and *TOPO* gene co-localisation. The first four samples are those shown in panels A-C, while the other six samples were run on other gels. TOR1 and TOR 4 gave identical results to TOR11, revealing that both chromosome IV homologues carry *SRA* in these three *Tbr* strains. LUMP 1198 and KETRI 2355 were the only *Tbr* examined that had a copy of *SRA* on a chromosome other than chromosome IV (black band); for KETRI 2355, *SRA* and *TOPO* did not co-localise.

To confirm this result, we separated chromosome-sized DNA molecules of different *Tbr* strains by pulsed field gel electrophoresis (PFG) and hybridised with *SRA* (Fig. 4). Although the molecular karyotypes of the *Tbr* strains differed markedly in number and size of chromosomal bands, in each strain *SRA* located to one or two chromosomes of about 2 Mb in size (Fig. 4B); the fainter hybridisation signals result from weak hybridisation with *SRA*-related *VSG* genes and hence can be disregarded. Sequential hybridisation of identical blots with chromosome-specific probes revealed that *SRA* co-localized with the gene for DNA topoisomerase on chromosome IV (Fig. 4C). The location of *SRA* on one or both copies of chromosome IV was confirmed for most of the other *Tbr* isolates tested (Fig. 4D), with the exception of KETRI 2355 for which another (unidentified) chromosomal band hybridised with *SRA* (Fig. 4D). For LUMP 1198, *SRA* hybridized with the compression zone (cz), a region of the gel where DNAs from several large chromosomes co-migrate, as well as chromosome IV (Fig. 4D). However, our subsequent analysis of *SRA* copy number and inheritance in crosses of LUMP 1198 demonstrated the presence of only a single *SRA* gene (see below), so we assume that the cz signal derived from *SRA*–related *VSG* genes rather than *SRA* itself.

Although the *SRA* gene and its immediate genomic environment have diverged in *Tbr* strains from northern and southern regions of East Africa [16,27], here *SRA* was located on chromosome IV in representative northern (TMRS 117) and southern (Gambella II, 058, EATRO 181) *Tbr* strains sequenced in the previous studies.

### Copy number of *SRA*

**Fig 5:**
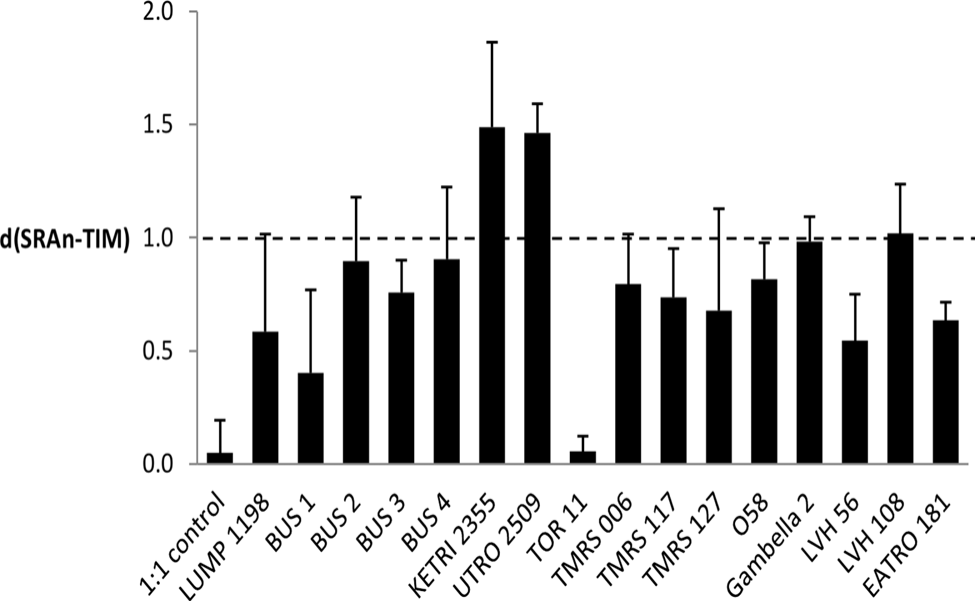
Copy number of *SRA* relative to *TIM*. Each bar shows the mean of 3 replicate experiments; bar is standard deviation. Dotted line at 1.0 indicates 1:2 ratio of *SRA* to *TIM*, while 1:1 ratio is at 0. C_t_ values for *SRA* were normalized (SRAn) using the standard curve obtained for the test plasmid containing one copy of each gene (S1 Fig), and then subtracted from *TIM* C_t_ values. The test plasmid was used as the 1:1 control.

The karyotype results suggest that *Tbr* strains generally have a single copy of *SRA*, or at most two copies. To verify this result, we estimated *SRA* copy number by quantitative PCR (qPCR) analysis, using copy number of the gene for triose phosphate isomerase (TIM) as the standard; in the diploid genome of *T*. *brucei* there are two copies of *TIM* [41]. The relative rates of amplification of *SRA* and *TIM* [ratio d(SRAnorm-TIM)], were calculated for genomic DNA from sixteen different *Tbr* strains using 3 replicates for each strain (Fig. 5). Most strains, including LUMP 1198 and KETRI 2355, had a ratio of approximately 1:2 SRA:TIM, except for *Tbr* TOR11, which had a ratio of approximately 1:1. This agrees with the karyotype analysis above, where most *Tbr* strains had a single chromosomal band hybridizing with *SRA*, except TOR11, which had two.

**Fig 6:**
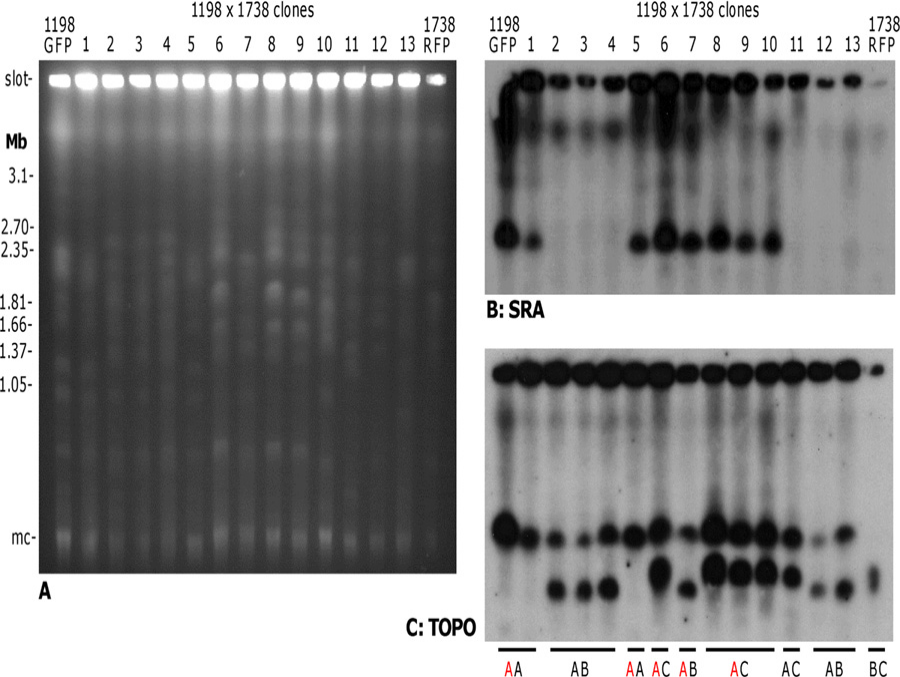
Inheritance of *SRA* in a cross of *T*. *b*. *rhodesiense* and *T*. *b*. *brucei*. A. Ethidium bromide stained gel comparing the molecular karyotypes of parental clones *T*. *b*. *rhodesiense* LUMP 1198 and *T*. *b*. *brucei* 1738 with those of 13 progeny clones; clones 1–5 and 6–13 are from two different tsetse flies, i.e. from two separate crosses. Size marker: chromosomal DNA from *Hansenula wingei*; mc = minichromosomes of 50–100 kb in size. B, C. Autoradiographs of blots of this PFG gel following hybridization with the probes indicated. Blots were washed to 0.1 × SSC at 65°C. Identification of chromosome IV homologues is shown below panel C: LUMP 1198 has homologues of the same size AA, while homologues of 1738 are designated B (lower) and C (upper); the red letter indicates the homologue carrying *SRA*. For hybrid clone 6, microsatellite analysis showed 3 alleles for a chromosome IV locus, indicating inheritance of both 1738 homologues.

The single, non-allelic copy of *SRA* in *Tbr* 058 and LUMP 1198 should segregate into 50% of hybrid progeny clones, assuming the rules of Mendelian inheritance are obeyed. Fig. 6 shows karyotype results for clones isolated from crosses of LUMP 1198 x 1738; the two chromosome IV homologues of *Tbr* LUMP 1198 co-migrate, but only one (red A) carries the *SRA* gene (Fig. 6B, lane 1). Three identical hybrid clones from cross 1198/1738-1 (lanes 2–4) lack *SRA* and are therefore assumed to have chromosome IV homologue A; these clones have also inherited the smaller chromosome IV homologue of 1738, B. Hybrid clones from cross 1198/1738-2 (lanes 6–13) demonstrate inheritance of parental chromosome IV homologues in all possible combinations (Fig. 6B, C).

**Fig 7:**
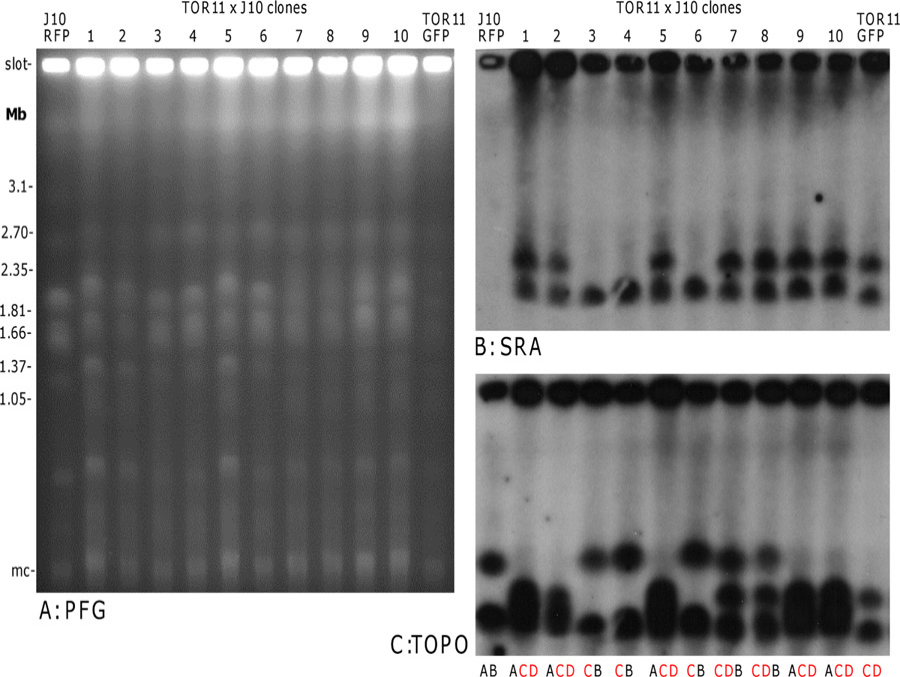
Inheritance of *SRA* in a cross of *T*. *b*. *rhodesiense* and *T*. *b*. *brucei*. A. Ethidium bromide stained gel comparing the molecular karyotypes of parental clones *T*. *b*. *rhodesiense* TOR11 and *T*. *b*. *brucei* J10 with those of 10 progeny clones; clones 1–6, 7–9 and 10 are from three different tsetse flies, i.e. from three separate crosses. Size marker: chromosomal DNA from *Hansenula wingei*; mc = minichromosomes of 50–100 kb in size. B, C. Autoradiographs of blots of this PFG gel following hybridization with the probes indicated. Blots were washed to 0.1 × SSC at 65°C. Identification of chromosome IV homologues is shown below panel C: J10 homologues are designated A (lower) and B (upper), while TOR11 homologues are designated C (lower) and D (upper) and both carry *SRA*, denoted by red letters.

In contrast, as *SRA* is located on both chromosome IV homologues in *Tbr* TOR11, diploid hybrid progeny from crosses with *Tbb* are expected to inherit a single copy of *SRA*. Results for crosses of TOR11 x J10 are shown in Fig. 7, where it can be seen that clones 3, 4 and 6 all have a single chromosome IV homologue carrying *SRA* from TOR11. However, all the other seven hybrid clones have both chromosome IV homologues with *SRA* from TOR11. Hybridisation intensities of individual chromosome bands suggest that these clones are trisomic for chromosome IV, with only one homologue from J10; this is obvious for clones 7 and 8, for which the chromosomal bands are well-separated (Fig. 7C); these clones also had three microsatellite alleles for the chromosome IV locus examined, confirming this result. Polyploid hybrids also occurred in the crosses involving *Tbr* 058 or LUMP 1198, explaining why a far greater proportion of hybrid clones than expected inherited *SRA* in these crosses (82%, 36 of 44 hybrid clones had *SRA*).

## Discussion

Our experimental crosses of *Tbr* and *Tbb* demonstrate unequivocally that the *SRA* virulence gene can be transferred by genetic exchange, thus creating new genotypes of potentially human infective parasites. The genetic heterogeneity of field isolates of *Tbr* from different regions of East Africa, together with their similarity to some *Tbb* isolates, first suggested that there might be hybridization between these two subspecies [42–44], and later studies have provided extensive evidence of genetic admixture [6,8].

Our crosses involved *Tbr* of the northern (LUMP 1198, TOR11) and southern (058) types [27], judged to differ in severity of HAT [45], and *Tbb* of different genotypic groups. *Tbb* J10 and 1738 belong to the kiboko/kakumbi group, distinguished from other East and West African *Tbb* such as Lister 427 by unusual isoenzymes, kinetoplast DNA maxicircle polymorphisms and microsatellite profiles [6,26,29]. Kiboko/kakumbi group isolates have never been found in human patients and originate from areas of East Africa that have a rich, large mammal fauna [46,47]. The tight association of kinetoplast and nuclear DNA polymorphisms suggested that the kiboko/kakumbi group circulates in separate wild animal-tsetse transmission cycles, without frequent sexual reproduction with other *Tbb*/*Tbr* strains. Contrary to this, we have shown that kiboko/kakumbi strains readily mate with different *Tbr*, as do other *Tbb* strains from both East and West Africa. Thus, there do not appear to be any intrinsic genetic barriers that prevent mating of *Tbr* and *Tbb*.

The accumulated data on location and copy number of *SRA* support the hypothesis that most *Tbr* strains have a single copy of *SRA* located in a *VSG* ES at the end of chromosome IV ([2,16] and this paper). As a consequence, *SRA* is only expressed when this ES is active, which means that the parasite is effectively restricted to use of this single ES in the human host. As noted above, a switch to another ES without *SRA* would be lethal for the trypanosome in a human host. This seems peculiar in a trypanosome that depends on antigenic variation for survival in the mammalian host and has multiple ES, especially considering that the *SRA* ES is truncated and lacks most *ESAG*’s [2]. How can we explain this? One possibility is that there are fitness costs associated with expression of *SRA* in other non-human mammalian hosts, though there is currently no evidence for this. *Tbr* is a zoonotic pathogen that arguably depends on a large population of non-human hosts for longterm persistence in endemic areas. Hence, the ability to easily switch off a single copy of *SRA* by swapping to *VSG* expression from another ES might be advantageous. Although it has been suggested that there are fitness costs associated with resistance to human serum in *Tbr* in tsetse [48], this seems unlikely; bloodstream form ES are silenced during trypanosome development in the insect vector, with activation of another set of specialized ES lacking *ESAG*’s in the infective metacyclics in the salivary glands [49]; therefore *SRA* is probably not expressed in the fly.

Our results suggest a more plausible hypothesis based on the dynamic between *Tbb* and *Tbr*. *SRA* is a truncated *VSG* gene [50,51] and is assumed to have evolved once, since the sequence and local genomic environment of *SRA* is conserved among different *Tbr* strains [16,27]. We do not know when this event occurred, but *SRA* would only have become advantageous when it allowed extension of *T*. *brucei’s* host range to include hosts with the trypanolytic factor, Apolipoprotein L1 (APOL1), in their serum [52]; this probably dates the evolution of *SRA*, and hence *Tbr*, to somewhere in the last 10 million years or so, when the ape lineages with APOL1 diverged [53,54]. Although *Tbr* might subsequently have been subject to selective pressure for gene or ES duplication, depending on how significant the size of the host population with APOL1, any increase in copy number of *SRA* would have been rapidly diluted by mating with *Tbb*. Currently, there are likely to be more *Tbb* than *Tbr* strains circulating in East Africa, considering the relative numbers of infected human and non-human hosts and the restricted distribution of *Tbr*. Hence the probability of mating between *Tbr* strains will be far lower than between *Tbb* and *Tbr*, except possibly in the midst of an epidemic. This may explain the duplication of *SRA* in TOR11 and other isolates TOR1 and TOR4 from the same HAT outbreak (S1 Table). We can assume that these isolates represent one *Tbr* strain that arose either by hybridization between *Tbr* strains or as a mutated strain with duplication of the *SRA* ES.

Since *Tbr* typically has only a single copy of *SRA* in a bloodstream form ES, metacyclics presumably do not express *SRA* when inoculated into the human host and will therefore not be protected from lysis by APOL1. Indeed, we were unable to demonstrate expression of *SRA* by RT PCR of RNA prepared from tsetse salivary glands infected with *Tbr* 058. *In vitro* experiments comparing the resistance of *Tbr* and *T*. *b*. *gambiense* (*Tbg1*) to lysis by human serum showed that few *Tbr* metacyclics, but the majority of *Tbg1* metacyclics, grew in medium containing human serum [55] and these authors hypothesized that survival of *Tbr* metacyclics in the human host depends on them being deposited in the skin tissue rather than bloodstream during tsetse bite, so that they are not directly exposed to the trypanolytic factor in the blood [55]. In support of this hypothesis, the absence of APOL1 in human tissue fluid needs to be verified.

In conclusion, new human infective strains of the human pathogen *Tbr* can be generated by recombination of *Tbr* with the much larger pool of animal-infective trypanosomes, *Tbb*. Such novel recombinants present a risk for future outbreaks of HAT.

## Supporting Information

S1 Fig Comparative efficiency of quantitative PCR (qPCR) for *SRA* and *TIM*. (DOCX)

S1 Table *Trypanosoma brucei rhodesiense* isolates; all are from human hosts and *SRA* positive. (DOCX)

S2 Table Primers used for quantitative PCR (qPCR). (DOCX)

S3 Table C_t_ values for qPCR of individual chromosomal bands of *T*. *b*. *rhodesiense* 058. (DOCX)

S4 Table C_t_ values for qPCR of individual chromosomal bands of *T*. *b*. *rhodesiense* LUMP 1198. (DOCX)
