# Supplementary material for: Genetic Recombination between Human and Animal Parasites Creates Novel Strains of Human Pathogen
Source: PLoS Negl Trop Dis. 2015 Mar 27;9(3):e0003665. doi: 10.1371/journal.pntd.0003665 (PMC4376878; doi:10.1371/journal.pntd.0003665)
Supplement: S1 Fig — (DOCX) [file pntd.0003665.s001.docx]

**Figure S1** Comparative efficiency of quantitative PCR (qPCR) for *SRA* and *TIM*.

A plasmid containing the *SRA* and *TIM* gene target regions in a 1:1 ratio was diluted in a ten-fold dilution series and used as template for qPCR using *SRA* or *TIM* primers. Standard curve using primers at 300 nM (*SRA*) and 500 nM (*TIM*) final concentrations.
