# Supplementary material for: Genetic Recombination between Human and Animal Parasites Creates Novel Strains of Human Pathogen
Source: PLoS Negl Trop Dis. 2015 Mar 27;9(3):e0003665. doi: 10.1371/journal.pntd.0003665 (PMC4376878; doi:10.1371/journal.pntd.0003665)
Supplement: S1 Table — (DOCX) [file pntd.0003665.s002.docx]

**Table S1** *Trypanosoma brucei rhodesiense* and *T. b. brucei* isolates; all *T. b. rhodesiense* isolates were from human hosts and *SRA* positive.

| **Isolate** | **Origin** | **Year** |
| --- | --- | --- |
| *T. b. rhodesiense* |  |  |
| Gambella II | Illubabor, Ethiopia | 1968 |
| LUMP 1198 | Busoga, Uganda | 1976 |
| KETRI 2355 | Busoga, Uganda | 1977 |
| UTRO 2509 | Busoga, Uganda | 1979 |
| UTRO 2516 | Busoga, Uganda | 1979 |
| BUS 1 | Busoga, Uganda | 1991 |
| BUS 2 | Busoga, Uganda | 1991 |
| BUS 3 | Busoga, Uganda | 1991 |
| BUS 4 | Busoga, Uganda | 1991 |
| TOR 1 | Tororo, Uganda | 1988 |
| TOR 4 | Tororo, Uganda | 1988 |
| TOR 11 | Tororo, Uganda | 1988 |
| LVH 56 | Lambwe valley, Kenya | 1978 |
| LVH 108 | Lambwe valley, Kenya | 1980 |
| TMRS 002 | Kasulu, Tanzania | 1991 |
| TMRS 006 | Kasulu, Tanzania | 1991 |
| TMRS 106 | Kibondo, Tanzania | 1994 |
| TMRS 108 | Kibondo, Tanzania | 1994 |
| TMRS 117 | Kibondo, Tanzania | 1994 |
| TMRS 119 | Kibondo, Tanzania | 1994 |
| TMRS 127 | Mpanda, Tanzania | 1994 |
| 058 | Luangwa valley, Zambia | 1974 |
| EATRO 181 | Botswana | 1980 |
| *T. b. brucei* |  |  |
| J10 Hyena | Luangwa valley, Zambia | 1973 |
| KETRI 1738 Sheep | Kiboko, Kenya | 1970 |
| 427 var 3 Sheep | Uganda | 1960 |
