# Supplementary material for: Genetic Recombination between Human and Animal Parasites Creates Novel Strains of Human Pathogen
Source: PLoS Negl Trop Dis. 2015 Mar 27;9(3):e0003665. doi: 10.1371/journal.pntd.0003665 (PMC4376878; doi:10.1371/journal.pntd.0003665)
Supplement: S2 Table — (DOCX) [file pntd.0003665.s003.docx]

**Table S2.** Primers used for quantitative PCR (qPCR)

| **Gene** | **Name** | **Sequence (5’→3’)** | **Chromosome location** |
| --- | --- | --- | --- |
| SRA | Q-SRA L | AGCCCGTCAAGAAGGTTT (18) | ? |
|  | Q-SRA R | GCTGAGATGTTGCTGTGG (18) |  |
| Triosephosphate isomerase TIM Tb927.11.5520 | Q-TIM L | AGTTGTCATCGCCTACGAAC (20) | XI |
|  | Q-TIM R | AGAACCGCCGTAAAGAATG (19) |  |
| Glucose-6-phosphate isomerase PGI Tb927.1.3830 | PGI L | TAAACATTGGCATCGGAG (18) | I |
|  | PGI R | TGCAAGAAGCAGAGGAAC (18) |  |
| α-Tubulin Tb927.1.2400 | TUB L | ACAAGACGATTGGCGTTGAG (20) | I |
|  | TUB R | CTGAAGACCAGTGCAG (16) |  |
| Trypanothione synthetase Tb927.2.4370 | TS L | GTTATATGAGGCCAAAGGGC (20) | II |
|  | TS R | AGGAAAGGCTTCTCTGGTTG (20) |  |
| Paraflagellar rod protein PFR1 Tb927.3.4290 | PFR1 L | CGGTGTGAGACAGATCTGAA (20) | III |
|  | PFR1 R | AAGAGAACCCAACGTGAGG (19) |  |
| DNA topoisomerase IB, large subunit Tb927.4.1330 | TOPO L | CGGAGCCGTATAAATGGTGT (20) | IV |
|  | TOPO R | CGTTTCTTCAGTTTCCCTCG (20) |  |
| Ribosomal RNA processing protein 6 RRP6 Tb927.4.1630 | RRP6 L | ACTAACAGACCCTCAGCGAG (20) | IV |
|  | RRP6 R | TCTGATCCAAACTTCACGG (19) |  |
| Lysosomal membrane protein P67 Tb927.5.1810 | P67 L | GGCTGATGCGTTACAACAAC (20) | V |
|  | P67 R | CGCCACAACAGCAATGAT (18) |  |
