# Supplementary material for: Genetic Recombination between Human and Animal Parasites Creates Novel Strains of Human Pathogen
Source: PLoS Negl Trop Dis. 2015 Mar 27;9(3):e0003665. doi: 10.1371/journal.pntd.0003665 (PMC4376878; doi:10.1371/journal.pntd.0003665)
Supplement: S3 Table — (DOCX) [file pntd.0003665.s004.docx]

**Table S3** C_t_ values for qPCR of individual chromosomal bands of *T. b. rhodesiense* 058.

| **Chr.** | **gene** | **A1** | **A2** | **A3** | **A4** | **A5** | **A6** | **A7** | **A8** |
| --- | --- | --- | --- | --- | --- | --- | --- | --- | --- |
| ? | SRA | 23.32 | 23.71 | 22.81 | 19.04 | **17.67** | 20.7 | 23.44 | 23.78 |
| I | GPI | 27.36 | 27.2 | 21.36 | 21.46 | 24.36 | **20.74** | 24.27 | 24.84 |
| I | TUB | 19.5 | 19.89 | **14.11** | **14.62** | 16.76 | **14.57** | 17.57 | 18.49 |
| II | TS | 24.72 | **20.18** | 22.89 | 22.89 | 24.1 | 26.04 | 26.23 | 26.51 |
| III | PFR1 | 26.83 | 26.45 | 23.57 | **19.5** | 22.17 | 23.17 | **20.9** | 21.86 |
| IV | TOPO | 22.94 | 22.86 | 19.98 | **16.77** | **17.16** | 19.54 | 22.19 | 22.27 |
| IV | RRP6 | 23.46 | 22.99 | 20.6 | **16.75** | **17.4** | 19.47 | 22.6 | 22.94 |
| V | P67 | 24.26 | 23.86 | 22.1 | **17.43** | **17.04** | 19 | 22.53 | 22.58 |

C_t_ values in bold type are the lowest for each chromosome.
