# Supplementary material for: Genetic Recombination between Human and Animal Parasites Creates Novel Strains of Human Pathogen
Source: PLoS Negl Trop Dis. 2015 Mar 27;9(3):e0003665. doi: 10.1371/journal.pntd.0003665 (PMC4376878; doi:10.1371/journal.pntd.0003665)
Supplement: S4 Table — (DOCX) [file pntd.0003665.s005.docx]

**Table S4** C_t_ values for qPCR of individual chromosomal bands of *T. b. rhodesiense* LUMP 1198.

| **Chr.** | **gene** | **B1** | **B2** | **B3** | **B4** | **B5** | **B6** | **B7** | **B8** |
| --- | --- | --- | --- | --- | --- | --- | --- | --- | --- |
| ? | SRA | 23.62 | 23.75 | 23.47 | 22.75 | **19.15** | 22.01 | 21.89 | 22.97 |
| I | GPI | 30.05 | 29.45 | 27.74 | 26.55 | **22.06** | 24.78 | **21.7** | 22.83 |
| I | TUB | 20.98 | 20.97 | 19.5 | 18.46 | **15.03** | 17.06 | **14.8** | 15.76 |
| II | TS | 28.19 | **22.97** | 25.07 | 24.49 | **22.2** | 25.81 | 25.76 | 26.49 |
| III | PFR1 | 28.16 | 28.31 | 26.49 | 23.82 | **20.78** | 23.68 | 24.67 | 25.63 |
| IV | TOPO | 24.17 | 24.14 | 23.06 | 22.14 | **18.42** | 21.11 | 21.02 | 22.25 |
| IV | RRP6 | 24.43 | 24.27 | 23.06 | 22.41 | **18.44** | 21.06 | 21.48 | 22.26 |
| V | P67 | 25.32 | 23.84 | **17.93** | **17.7** | 20.7 | 22.97 | 22.93 | 23.2 |

C_t_ values in bold type are the lowest for each chromosome.
